# Case report: Complex evaluation of coagulation, fibrinolysis and inflammatory cytokines in a SARS-CoV-2 infected pregnant woman with fetal loss

**DOI:** 10.3389/fimmu.2024.1329236

**Published:** 2024-02-21

**Authors:** Eszter Lilla Tóth, Rita Orbán-Kálmándi, Zsuzsa Bagoly, Linda Lóczi, Tamás Deli, Olga Török, Sarolta Molnár, Sándor Baráth, Parvind Singh, Zsuzsanna Hevessy, Éva Katona, Miklós Fagyas, Attila Ádám Szabó, Szabolcs Molnár, Zoárd Tibor Krasznai

**Affiliations:** ^1^ Department of Obstetrics and Gynecology, Faculty of Medicine, University of Debrecen, Debrecen, Hungary; ^2^ Doctoral School of Molecular Medicine, University of Debrecen, Debrecen, Hungary; ^3^ Division of Clinical Laboratory Sciences, Department of Laboratory Medicine, Faculty of Medicine, University of Debrecen, Debrecen, Hungary; ^4^ Kálmán Laki Doctoral School, University of Debrecen, Debrecen, Hungary; ^5^ Department of Pathology, Faculty of Medicine, University of Debrecen, Debrecen, Hungary; ^6^ Department of Laboratory Medicine, Faculty of Medicine, University of Debrecen, Debrecen, Hungary; ^7^ Division of Clinical Physiology, Department of Cardiology, Faculty of Medicine, University of Debrecen, Debrecen, Hungary

**Keywords:** COVID-19, fetal death, hemostasis, placenta, case report, stillbirth

## Abstract

**Background:**

SARS-CoV-2 infection during pregnancy increases the risk of severe obstetrical complications. Detailed evaluation of COVID-19-associated coagulopathy in a pregnancy with stillbirth hasn’t been described so far. Besides knowledge gaps in the pathomechanism leading to stillbirth in COVID-19 pregnancies, currently, no prognostic biomarker is available to identify pregnant patients who are at imminent risk of COVID-19-associated maternal and fetal complications, requiring immediate medical attention.

**Case:**

Here we report the case of a 28-year-old SARS-CoV-2 infected pregnant patient, admitted to our hospital at 28 weeks of gestation with intrauterine fetal loss. The presence of SARS-CoV-2 placentitis was confirmed by immunohistological evaluation of the placenta. She had only mild upper respiratory symptoms and her vital signs were within reference throughout labor and postpartum. The stillborn infant was delivered per vias naturales. Fibrinogen concentrate was administered before and after labor due to markedly decreased fibrinogen levels (1.49 g/l) at admission and excessive bleeding during and after delivery. Although coagulation screening tests were not alarming at admission, the balance of hemostasis was strikingly distorted in the patient. As compared to healthy age- and gestational age-matched pregnant controls, increased D-dimer, low FVIII activity, low FXIII level, marked hypocoagulability as demonstrated by the thrombin generation assay, together with shortened clot lysis and decreased levels of fibrinolytic proteins were observed. These alterations most likely have contributed to the increased bleeding observed during labor and in the early postpartum period. Interestingly, at the same time, only moderately altered inflammatory cytokine levels were found at admission. Serum ACE2 activity did not differ in the patient from that of age- and gestational age-matched healthy controls, suggesting that despite previous speculations in the literature, ACE2 may not be used as a potential biomarker for the prediction of COVID-19 placentitis and threatening fetal loss in SARS-CoV-2-infected pregnancies.

**Conclusions:**

Although based on this case report no prognostic biomarker could be identified for use in pregnant patients with imminent risk of fetal loss associated with COVID-19 placentitis, the above-described hemostasis alterations warrant awareness of postpartum hemorrhagic complications and could be helpful to identify patients requiring intensified medical attention.

## Introduction

SARS-CoV-2 infection during pregnancy increases the risk of severe obstetrical complications, including preterm birth, preeclampsia, placental abruption or stillbirth (1–3). Despite the favorable evolution of the COVID-19 pandemic, the virus continues to circulate and awareness is suggested in vulnerable groups, including immunocompromised and pregnant individuals (4). The pathomechanism leading to stillbirth in COVID-19 pregnancies hasn’t been unraveled, moreover, currently, no prognostic biomarker is available to identify pregnant patients who are at imminent risk of COVID-19-associated maternal and fetal complications, requiring immediate medical attention.

This case presentation illustrates detailed hemostasis alterations, inflammatory cytokine levels, and angiotensin-converting enzyme 2 (ACE2) levels in a COVID-19-infected woman experiencing pregnancy loss and demonstrating SARS-CoV-2 placentitis at 28 weeks of gestation.

## Methods

### Blood sampling, processing and general laboratory tests

Venous blood was drawn from the patient at admission and from 10, age-and gestational-age matched, healthy, SARS-CoV-2 negative control pregnant women. Exclusion criteria of healthy pregnant women included history of major illnesses (hypertension, diabetes, autoimmune disease, malignancy, thrombophilia or hemorrhagic diathesis), pregnancy complications, no consent. Six healthy controls were vaccinated against SARS-CoV-2, all administered before pregnancy. Routine laboratory tests (complete blood count, liver and renal function tests, C-reactive protein measurement) were carried out by standard methods. Coagulation screening tests (prothrombin time, activated partial thromboplastin time, thrombin time) and fibrinogen were measured by routine methods (Siemens Healthcare Diagnostic Products, Marburg, Germany). ACE and ACE2 activities were assessed using specific quenched fluorescent substrates as described earlier (5, 6). Anti-SARS-CoV-2 rapid antigen test was carried out in the patient and controls from nasopharyngeal swab samples (Genedia, St. Ingbert, Germany). For the quantitative determination of antibodies against SARS-CoV-2 nucleocapsid (N) and spike (S) proteins, Elecsys^®^ anti-SARS-CoV-2 tests were used (Roche Diagnostics, Mannheim, Germany).

### Comprehensive assessment of coagulation and fibrinolysis

Quantitative D-dimer levels were measured using a particle-enhanced, immuno-turbidimetric assay (Siemens Healthcare Diagnostic Products, Marburg, Germany). Factor VIII (FVIII) activity, von Willebrand factor (VWF) antigen levels, α2-plasmin inhibitor (α2-PI) and plasminogen activities were determined by standard methods as described previously (7, 8). Thrombin generation (TG) test was carried out using the Thrombinoscope CAT assay (Calibrated Automated Thrombogram, Maastricht, The Netherlands) according to the manufacturer’s instructions (9). A sandwich ELISA assay was used to determine the levels of plasma factor XIII (FXIII-A_2_B_2_) antigen levels (10) as well as total FXIII-B subunits (11). Clot lysis assay (CLA) was performed as previously described (12). Plasminogen activator inhibitor-1 (PAI-1) antigen levels were measured using Technozym PAI-1 Antigen ELISA assay (Technoclone, Vienna, Austria) (13).

### Analysis of inflammatory cytokines/chemokines

Cytokine profiling was carried out using a bead-based multiplex fluorescent immunoassay (LEGENDplex™ Human Inflammation Panel, BioLegend, San Diego, CA) according to the manufacturer’s instructions. The panel allowed simultaneous quantification of 13 human inflammatory cytokines (IL1-β, IFN-α2, IFN-γ, TNF- α, MCP-1, IL-6, IL-8, IL-10, IL-12p70, IL-17A, IL-18, IL-23, IL-33). The samples were read using BD FACS Canto II flow cytometer (BD Biosciences, San Jose, CA, USA), and the data were analyzed using LEGENDplex™ Data Analysis Software V8.0 (BioLegend).

### Histopathological analysis of the placenta

A detailed technical description of the histopathological analysis of the placenta is provided as **Supplementary Material**. Briefly, after formalin fixation and paraffin embedding of tissue blocks, specimens were sectioned. Besides standard hematoxylin and eosin stained slides, immunohistological evaluation was performed for CD68 (Dako, Agilent Technologies Company, Santa Clara, CA) and anti-SARS-CoV-2 spike protein staining (Cell Signalling Technology, Danvers, MA) followed by visualization with the UltraView and OptiView DAB IHC Detection kits (Ventana Medical Systems, Oro Valley, AZ).

### Informed consent

Informed consent was obtained from the patient and the healthy control pregnant individuals. Approval was obtained by the Institutional Ethics Committee of the University of Debrecen and the Ethics Committee of the National Medical Research Council (IV/3267-3/2021/EKU).

## Results

### Case description

A 28-year-old woman was admitted to our hospital at 28 weeks of gestation due to a day history of decreased fetal movement and mild abdominal cramps in the past 2 hours. She was admitted during the 4th wave of COVID-19 (November, 2021), mainly dominated by the SARS-CoV-2 Delta (B.1.617.2) variant. At admission, anti-SARS-CoV-2 rapid antigen test was performed with a positive result. The patient did not receive vaccination against SARS-CoV-2, nor had previous COVID-19 disease. Medical history of the patient included transcervical fibroid and endometrial polyp resection by hysteroscopy in 2016, a loop electrosurgical excision procedure in 2017 with negative histology. No other relevant medical, family or genetic past histories were elicited. The patient’s obstetric history included one previous pregnancy delivered at term via cesarean section. The current pregnancy was uneventful until admission. She was not taking any medications and denied smoking or alcohol during the pregnancy. Obstetric examination including ultrasound revealed intrauterine fetal loss, with an estimated gestational age of 28 weeks and 1 day. Clinical or ultrasound signs suggestive of placental abruption or preterm rupture of the membranes were not detected. At admission, the uterine cervix was closed, and labor had not started, minor uterine contractions were present. She had mild upper respiratory symptoms and was classified as having an overall mild illness (stage II) according to the COVID-19 disease severity classification of the National Institutes of Health (14). Her vital parameters were stable and showed only minor alterations from the reference: pulse was 88 beats/min, blood pressure was 160/100 mmHg, body temperature was 36.2 °C, respiratory rate was 18 breaths/min, and the oxygen saturation was 99% without supplemental O_2_ therapy at admission. Baseline clinical and laboratory parameters of the patient as compared to healthy age- and gestational age-matched controls are summarized in **Table 1**.

**Table 1:** Clinical and laboratory characteristics of the patient at admission as compared to age- and gestational week-matched healthy pregnant controls.

Variables	Patient	Healthy pregnant controls (n=10)
Age, years	28	28 ± 1.2
BMI, kg/m^2^	24.0	31 ± 4.2
Gestational weeks	28	28 ± 1
Blood count		
WBC, G/l	3.5	8.6 ± 3.1
Neutrophils, G/l	2.1	6.5 ± 2.0
Eosinophils, G/l	0.01	0 ± 0.04
Lymphocytes, G/l	1.2	0.9 (0.6-1.2)
Monocytes, G/l	0.1	0.7 ± 0.2
RBC, T/l	4.4	3.9 ± 0.3
Hemoglobin, g/l	133	120 ± 11
Platelet count, G/l	77	253 ± 34
Chemistry parameters		
AST, U/l	67	20 ± 3
LDH, U/l	628	183 ± 28
ALT, U/l	64	22 (11-50)
γGT, U/l	82	22 ± 6
Total bilirubin, μmol/l	14.4	7 ± 4.2
Serum glucose, mmol/l	6.6	5 ± 0.6
hsCRP, mg/l	14.9	4.6 (1.7-8.4)
Creatinine μmol/l	49	45 ± 12
Urea mmol/l	2.4	2.5 ± 0.6
Screening tests of hemostasis		
PT, sec	8.6	8 ± 0.3
aPTT, sec	32.1	29 ± 6.9
TT, sec	20.4	16 ± 1.2
Angiotensin-converting enzyme (ACE) levels		
ACE, U/l	10.0	8.5 ± 2.7
ACE2, mU/l	21.7	23.7 ± 3.9
Anti-SARS-CoV-2 seropositivity	Patient	Limit of reference
Anti-SARS-CoV-2 N total Ig (IgG/M), COI	8.8	≥ 1.0
Anti-SARS-CoV-2 S total Ig (IgG/IgM), BAU/ml	16.3	≥ 0.8
Anthropometric parameters	Fetus	Reference*
Weight, g	915	967-1383
HC, mm	250	247-280
AC, mm	175	220-260

Continuous variables are expressed as mean ± SD or median (IQR). AC, abdominal circumference; ACE, angiotensin-converting enzyme; ALT, alanine aminotransferase; anti-SARS-CoV-2 N, anti-SARS-CoV-2 nucleocapsid antibody; anti-SARS-CoV-2 S, anti-SARS-CoV-2 spike protein antibody; aPTT, activated partial thromboplastin time; AST, aspartate aminotransferase; BMI, body mass index; COI, cut-off index; γGT, gamma-glutamyl transferase; HC, head circumference; hsCRP, high-sensitivity C-reactive protein measurement; LDH, lactate dehydrogenase; n, number; PT, prothrombin time; SARS-CoV-2, severe acute respiratory syndrome coronavirus 2; TT, thrombin time; RBC, red blood cell count; WBC, white blood cell count.

*Based on Kiserud et al. (15).

Due to the previous cesarean delivery in her history, labor was induced with the use of Foley balloon catheter, amniotomy and oxytocin infusion. Before the induction of labor, intravenous methylprednisolone (40 mg) was administered and blood was taken for the specific assessment of hemostasis and inflammatory cytokines. During induction of labor, oral antibiotics (amoxicillin and clavulanic acid, 875/125 mg), and highly purified human fibrinogen concentrate (2 g, Fibryga, Octapharma) were provided as prophylaxis to ensure a fibrinogen level ≥1.5 g/l. The amniotic fluid was meconium-stained. Less than six hours later the patient delivered the stillborn infant per vias naturales weighing 915 g without signs of fetal maceration (**Table 1**). After delivery, placental retention and uterine scar dehiscence was excluded by manual uterine revision. One hour after delivery heavy vaginal bleeding occurred, therefore, additional 2 grams of fibrinogen concentrate was administered. Uterine atony was excluded, and oxytocin was administered as prophylactic uterotonic. Signs of heavier bleeding diminished within 20 min, and no further medication was needed. Vital signs showed no considerable change during labor and early postpartum. The stillborn infant and the placenta with the umbilical cord were subjected to histopathologic examination. The patient was discharged after three days and low molecular weight heparin prophylaxis was provided for 10 days. Bromocriptine was prescribed for ablactation and psychological counseling was offered. The late postpartum period was uneventful, no thrombotic or hemorrhagic event occurred.

### Comprehensive analysis of coagulation, fibrinolysis and inflammatory cytokines

Laboratory tests at admission revealed slight leukopenia and thrombocytopenia, but hemoglobin concentration was within the normal range. As compared to healthy pregnant controls, elevated hepatic transaminases, bilirubin, lactate dehydrogenase (LDH) and mildly increased C-reactive protein (CRP) levels were observed, while kidney function was unaltered (**Table 1**). Coagulation screening tests, ACE and ACE2 activities showed no relevant alterations from controls at admission. Anti-SARS-CoV-2 testing revealed low-titer seropositivity for anti-SARS-CoV-2 anti-nucleocapsid and anti-spike total Ig (IgG/IgM) suggestive of recent seroconversion. Repeated testing of routine laboratory parameters within the first day after labor indicated a mild reduction in hemoglobin levels (119 g/l), an increase in platelet count (104 G/l), and in white blood cell count (6.45 G/l). After giving birth, hepatic transaminases including LDH showed a decrease, while CRP levels remained similar to that observed at admission (12.7 mg/l).

**Figure 1:**
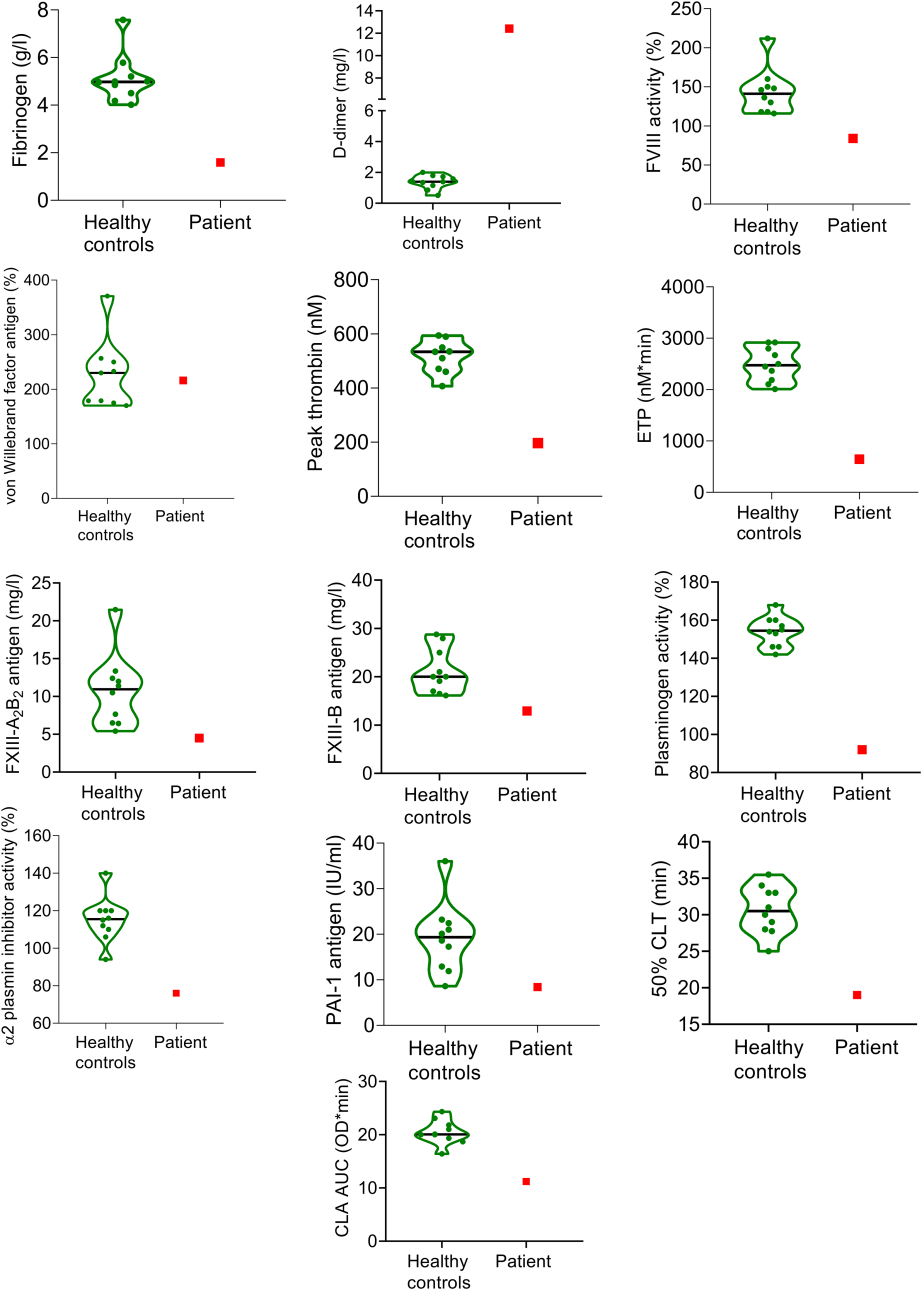
Markers of coagulation and fibrinolysis in the SARS-CoV-2 infected pregnant patient at admission and in healthy, age- and gestation-week matched pregnant controls (n=10). The patient’s results are indicated with red squares, while green violin plots show the results of healthy controls. Black horizontal lines indicate median values of controls.

Results of the comprehensive analysis of coagulation and fibrinolysis in the patient at admission are shown in **Figure 1**. Although the screening tests of coagulation were unremarkable at admission, the balance of hemostasis was strikingly distorted in the patient. As compared to healthy age- and gestational age-matched pregnant controls, the patient presented with markedly decreased fibrinogen levels (1.49 g/l), increased D-dimer (12.4 mg/l), low FVIII activity (84%), low FXIII levels (FXIII A_2_B_2_ antigen: 4.5 mg/l, FXIII-B antigen: 12.93 mg/l). The extent of TG was unusually low in the patient (peak thrombin: 196 nM; ETP: 646 nM*min), although she was not using anticoagulants a this time. Moreover, low plasminogen activity (92%), low α2-PI level (76%), and shortened clot lysis (50% clot lysis time: 19 min; area under the curve: 11.2 OD*min) were observed in the patient’s plasma sample at admission as compared to that of healthy control pregnancies.

**Figure 2:**
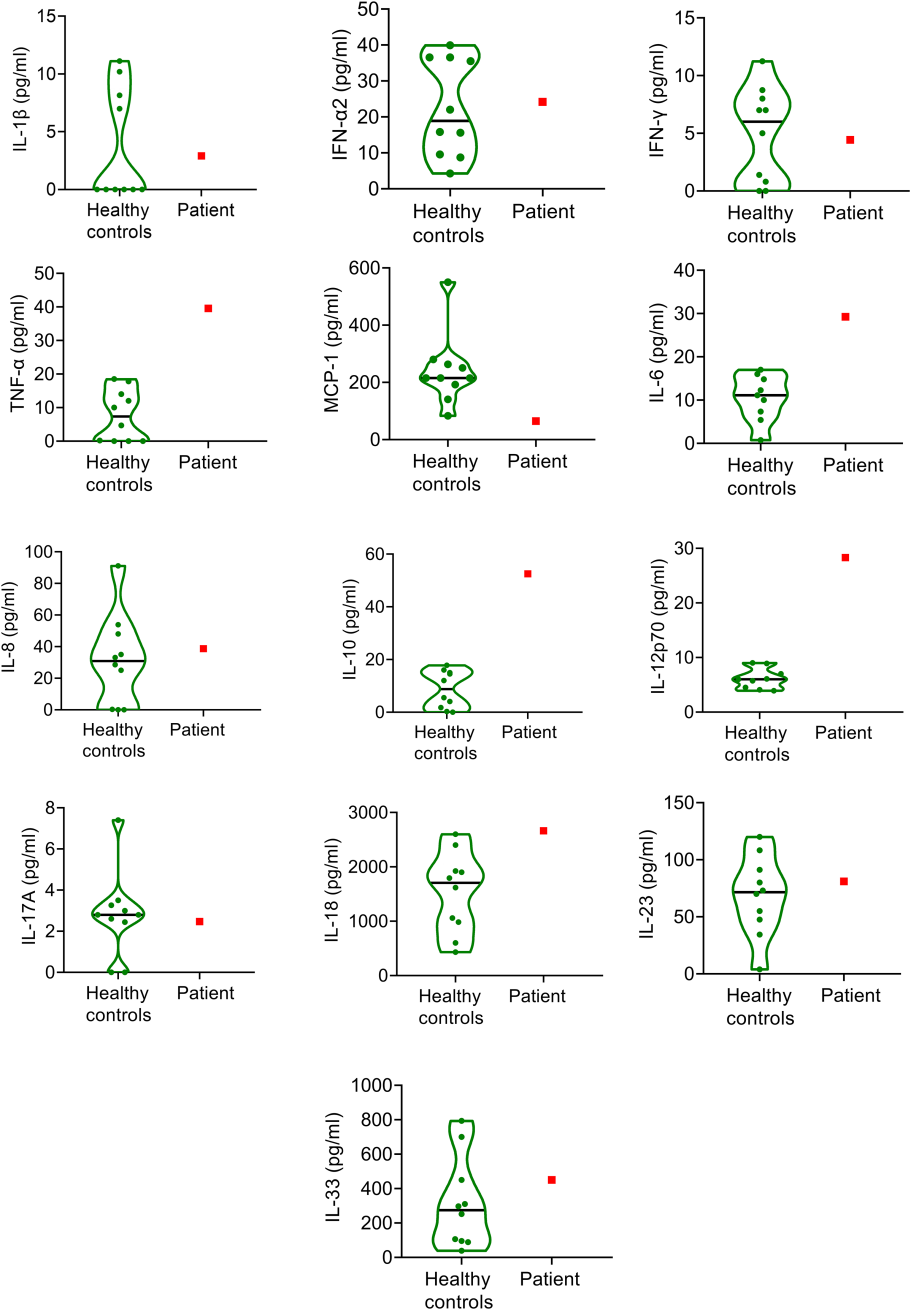
Inflammatory cytokine levels in the SARS-CoV-2 infected pregnant patient at admission and in healthy, age- and gestation-week matched pregnant controls (n=10). The patient’s results are indicated with red squares, while green violin plots show the results of healthy controls. Black horizontal lines indicate median values of controls.

Despite these marked changes in the balance of hemostasis and fibrinolysis, a comprehensive analysis of inflammatory cytokines from the patient’s serum sample of the same time point (**Figure 2**) showed normal IL-1β, IFN-α2, IFN-γ, IL-8, IL-17A, IL-23 and IL-33 levels, overlapping with the results of healthy control pregnant patients. As expected, increased levels of the proinflammatory cytokines, IL-6, TNFα, IL-12p70, and IL-18 were detected, while the level of IL-10, an anti-inflammatory cytokine was also elevated in the patient. Overall, the inflammatory cytokine profile was suggestive of the acute-subacute phase of viral infection, but the extent of increase did not indicate extensive virus-induced cytokine production or cytokine storm as seen in the case of severe COVID-19 diseases. This was in line with the mild respiratory symptoms of the patient.

### Histopathological examination of the placenta

**Figure 3:**
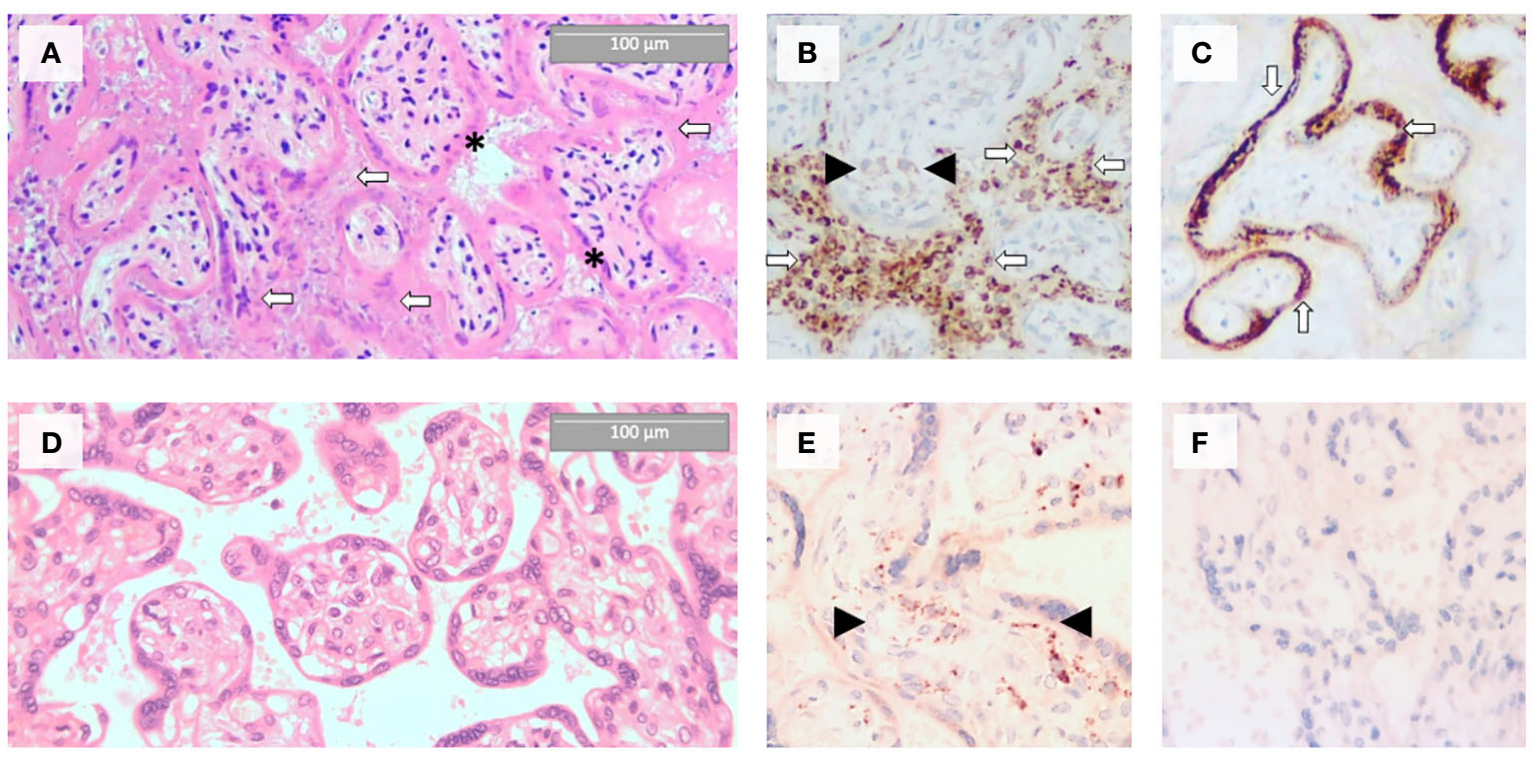
Histopathological evidence of COVID-19 placentitis in the placental tissue of the stillborn fetus **(A–C)** as compared to the morphology of a control healthy term placenta **(D–F)**. **(A)** Massive perivillous fibrin deposition (white arrows) and trophoblast cell necrosis (black asterisks) by hematoxylin and eosin staining. **(B)** Abundance of CD68 immunopositive histiocytes in the intervillous spaces (white arrows, anti-CD68 staining). Black arrowheads indicate Hofbauer cells. **(C)** Intensive SARS-CoV-2 spike protein immunopositivity of the syncytiotrophoblast (white arrows, anti-SARS-CoV-2 spike protein staining). **(D)** HE staining of a healthy term placenta with normal morphology. **(E)** In healthy term placenta, CD68 positive Hofbauer cells (black arrowheads) are present in the villus stroma, but intervillous spaces are free of histicocytes (anti-CD68 staining). **(F)** In healthy term placenta, SARS-CoV-2 spike is not expressed (anti-SARS-CoV-2 spike protein staining).

Anthropometric parameters of the stillborn infant are listed in **Table 1**. The placenta weighed 165 g (10-25 percentile for gestational age) and had an excentric three-vessel cord with a 3 cm long velamentous vessel, without any rupture. The cord was 41 cm long, and 1.5 cm wide, its coiling index was slightly below normal (3.5 coil/41 cm). The fetal surface of the placenta and the fetal membranes were smooth. The maternal surface was intact. Placental consistency was slightly more firm than average, resembling a rubber gum-like consistency. Cut sections demonstrated diffuse greyish-red deposits with multiple areas of dark red spots. One peripheral focus of an obsolete infarct was found, occupying approximately 1% of the placenta. Microscopic findings included diffuse syncytiotrophoblast necrosis, massive perivillous fibrin deposition and diffuse intervillositis with a mixture of inflammatory cells, dominantly neutrophils surrounded by hystiocytes (**Figure 3A**). Fibrin and inflammatory cells obliterated the intervillous spaces. Chronic histiocytic intervillositis was confirmed by CD68 immunohistochemistry (**Figure 3B**). To confirm the presence of COVID-19 placentitis, anti-SARS-CoV-2 spike protein immunohistochemistry was applied. The presence of viral protein was demonstrated in the placental tissue, mostly in the cytoplasm of trophoblast cells (**Figure 3C**), for comparison, the histopathological examination of a healthy control placenta is demonstrated on **Figures 3D–F**.

## Discussion

To the best of our knowledge, here we report the first case of detailed hemostasis, fibrinolysis and inflammatory cytokine analysis together with the histopathological examination of the placenta in a pregnancy with SARS-CoV-2 infection and intrauterine fetal loss. Although stillbirth is a rare, but well-known complication of COVID-19, the pathomechanism has not been fully elucidated as yet. In this case, although it is impossible to justify unequivocally, the most probable cause of intrauterine demise was SARS-CoV-2 placentitis, which has been associated with stillbirth or miscarriage in as high as 50% of detected cases. Other potential causes, e.g. velamentous cord insertion described in the histopathological examination of the placenta cannot be fully ruled out, however, based on a meta-analysis this variation itself only increases the risk of stillbirth by 0.8% (16). According to recent data, SARS-CoV-2 placentitis plays an important role in the pathological events leading to intrauterine death, due to a combination of concurrent destructive mechanisms including increased fibrin deposition, chronic hystiocytic intervillositis and trophoblast necrosis (17, 18). An important observation is that in all reported cases of SARS-CoV-2 placentitis causing stillbirth, mothers were unvaccinated, just as in this case (17). The lack of vaccination or previous infection have been described as an important contributor to maternal viremia and vertical transmission (17). Another important finding to highlight is the lack of evidence that perinatal death is induced by the direct viral infection of fetal organs, rather, placental insufficiency and consequent fetal hypoxemic injury of the fetus results in demise. Little is known about which SARS-CoV-2 variant is more likely to cause placentitis and stillbirth. Limited evidence points toward the Delta variant causing greater viral load, that more likely results in viremia and vertical transmission (19). Another interesting and yet unexplained observation from our and previous cases is the limited correlation between maternal COVID-19 severity and the placental infection resulting in insufficiency and stillbirth (17). To date, detailed hemostasis investigations have not been published in such cases, as yet. Here we report a considerably distorted hemostasis balance within an estimated 1 day of intrauterine death, accompanied by only moderately altered inflammatory cytokine levels. During a healthy pregnancy, pro-inflammatory cytokines (e.g. IL-6, TNF-α, etc.) become elevated, contributing to normal immune adaptations and fetal development, while anti-inflammatory cytokines, (e.g. IL-10, etc.) are also increased to prevent excessive inflammatory responses (20). It has been shown that inflammatory cytokines/chemokines are significantly dysregulated in mothers infected with SARS-CoV-2 prior to delivery, but uninfected pregnant controls may also exhibit a large degree of variability in their cytokine/chemokine levels (21). In our case, as expected, increased proinflammatory cytokine levels, e.g. IL-6, TNFα, IL-12p70, and IL-18 were detected, together with the anti-inflammatory IL-10. Other tested cytokine/chemokine levels showed an overlap with that observed in healthy pregnancies, which may be attributed to the mild infection of the patient and the variable cytokine/chemokine profile of pregnant controls.

Although the results of coagulation screening tests were not alarming, extensive measurements of specific coagulation and fibrinolysis markers showed marked hypocoagulability and signs of hyperfibrinolysis indicative of a bleeding phenotype. Notably, low fibrinogen levels, remarkably low TG parameters, low FVIII levels, together with increased D-dimer and shortened clot lysis, decreased levels of fibrinolytic proteins were found, which, altogether most likely have contributed to the increased bleeding observed during labor and in the early postpartum period. The patient had thrombocytopenia and elevated liver transaminases, reminiscent of HELLP syndrome (hemolysis, elevated liver enzymes, low platelet count), but a slightly altered, untypical presentation was observed. This type of presentation of HELLP, i.e. the absence of proteinuria, and extensive hemolysis has been described earlier in COVID-19-associated pregnancies (22). The presence of this type of HELLP, together with the alterations in the balance of hemostasis and fibrinolysis described in this patient warrants future investigations. The findings of hypofibrinogenaemia have been also described earlier in pregnancies with COVID-19, which differs vastly from the COVID-19 associated coagulopathy in non-pregnant individuals, where fibrinogen levels are usually within the reference range or even increased (23). Given the association between hypofibrinogenaemia and postpartum hemorrhage, awareness is needed when it comes to the management of SARS-CoV-2-infected pregnant women, even if they are asymptomatic or have only mild respiratory symptoms (24, 25). Aside from our report, detailed analysis of coagulation and fibrinolysis has not been published in women with COVID-19 and intrauterine fetal loss, and the prognostic significance of these markers has not been evaluated. Importantly, assessment of such detailed hemostasis investigations was helpful in deciding on the potential hypo-or hypercoagulable state caused by COVID-19 infection in the patient. Despite the comprehensive evaluations carried out on this patient, no candidate marker seems to stand out as a feasible test for the prediction of threatening pregnancy loss. ACE2, the main receptor for SARS-CoV-2 in human placentas has been indicated as the key entry door to viral transmission (26). In earlier reports, ACE2 has been described as a potential marker of obstetrical complications in SARS-CoV-2-infected pregnant women (27, 28). In placentas infected by SARS-CoV-2, ACE2 is down-regulated, which can potentially alter key physiological processes during placental development and vascularization (27, 28). Moreover, the loss of ACE2 function leads to a decrease in plasma levels of angiotensin (1–4, 17, 29, 30), potentiating vasoconstriction, hypercoagulation, which has been implicated to play a role in obstetrical complications (31). Based on this, the circulating levels of ACE2 has been proposed as a promising potential biomarker for the prediction of COVID-19 placentitis and threatening fetal loss in SARS-CoV-2-infected pregnancies. However, this hypothesis was not confirmed in this case, as circulating ACE2 levels did not differ in this patient compared to healthy pregnant women. Studies also pointed out that although ACE2 may paradoxically act as a risk factor for viral transmission, it may also act as a protective factor by preserving physiological adaptations in COVID-19-exposed pregnancies (27). Such intriguing questions warrant future research.

Limitations. A limitation of the presented study lies in the inherently retrospective nature of the analysis, as it relies on *post-hoc* examination and interpretation of data. Although data presented here suggests probable SARS-CoV-2 vertical transmission, detection of the presence of SARS-CoV-2 in the placenta or fetal tissues by means of other methods was not available, moreover, the cause of stillbirth might have been multi-factorial. Due to the evolving nature of COVID-19 and its variants, the generalizability of the findings may be restricted.

## Conclusion

In this case of a 28-year-old pregnant patient with confirmed acute SARS-CoV-2 infection and intrauterine fetal loss, comprehensive analysis of hemostasis and inflammatory cytokines indicated a distorted coagulation balance with hypofibrinogenemia, marked hypocoagulability, altered fibrinolytic balance, accompanied with only mild inflammatory cytokine responses and normal ACE2 levels. It is important to highlight that COVID-19-associated coagulopathy had distinct features in this patient as compared to non-pregnant patients, and coagulation screening tests were unremarkable. Although increased D-dimer is a usual presentation of COVID-19 associated coagulopathy, low fibrinogen levels and hypocoagulability, as confirmed by the TG assay, are not typical for COVID-19 in non-pregnant cases. These results, however, particularly hypofibrinogenemia and low TG have high predictive value for postpartum bleeding complications. Based on this case, early administration of fibrinogen concentrate is useful to overcome severe hemorrhage during and after labor. On the other hand, based on this case, potential markers predicting fetal loss in pregnancies with COVID-19 cannot be derived. Nevertheless, the above-described hemostasis alterations warrant awareness in SARS-CoV-2-infected pregnancies and could be useful to identify patients requiring intensified medical attention.

## Data availability statement

The raw data supporting the conclusions of this article will be made available by the authors, without undue reservation.

## Ethics statement

The studies involving humans were approved by Institutional Ethics Committee of the University of Debrecen and the Ethics Committee of the National Medical Research Council. The studies were conducted in accordance with the local legislation and institutional requirements. The participants provided their written informed consent to participate in this study. Written informed consent was obtained from the individual(s) for the publication of any potentially identifiable images or data included in this article.

## Author contributions

ET: Data curation, Formal analysis, Investigation, Methodology, Writing – original draft. RO: Data curation, Writing – original draft, Formal analysis. ZB: Conceptualization, Data curation, Investigation, Methodology, Supervision, Validation, Writing – original draft, Writing – review & editing. LL: Data curation, Formal analysis, Investigation, Writing – review & editing. TD: Writing – review & editing. OT: Writing – review & editing. SaM: Writing – review & editing. SB: Writing – review & editing. PS: Writing – review & editing. ZH: Writing – review & editing. ÉK: Writing – review & editing. MF: Writing – review & editing. AS: Writing – review & editing. SzM: Writing – review & editing. ZK: Conceptualization, Funding acquisition, Investigation, Resources, Supervision, Writing – original draft.
