# Supplementary material for: Case report: Complex evaluation of coagulation, fibrinolysis and inflammatory cytokines in a SARS-CoV-2 infected pregnant woman with fetal loss
Source: Front Immunol. 2024 Feb 21;15:1329236. doi: 10.3389/fimmu.2024.1329236 (PMC10915050; doi:10.3389/fimmu.2024.1329236)
Supplement: Supplementary file 1 [file DataSheet_1.docx]

Supplementary Material

Case report: Complex evaluation of coagulation, fibrinolysis and inflammatory cytokines in a SARS-CoV-2 infected pregnant woman with fetal loss

**Eszter Lilla Tóth^1,2^, Rita Orbán-Kálmándi^3^, Zsuzsa Bagoly^3^, Linda Lóczi^3,7^, Tamás Deli^1^, Olga Török^1^, Sarolta Molnár^5,^ Sándor Baráth^4^, Parvind Singh^4,7^, Zsuzsanna Hevessy^4^, Éva Katona^3^, Miklós Fagyas^6^, Attila Ádám Szabó^6,7^, Szabolcs Molnár^1^, Zoárd Tibor Krasznai^1^***

^1^ Department of Obstetrics and Gynecology, Faculty of Medicine, University of Debrecen, Debrecen, Hungary

^2^ Doctoral School of Molecular Medicine, University of Debrecen, Debrecen, Hungary

^3^ Division of Clinical Laboratory Sciences, Department of Laboratory Medicine, Faculty of Medicine, University of Debrecen, Debrecen, Hungary

^4^ Department of Laboratory Medicine, Faculty of Medicine, University of Debrecen, Debrecen, Hungary

^5^ Department of Pathology, Faculty of Medicine, University of Debrecen, Debrecen, Hungary

^6^ Division of Clinical Physiology, Department of Cardiology, Faculty of Medicine, University of Debrecen, Debrecen, Hungary

^7^ Kálmán Laki Doctoral School, University of Debrecen, Debrecen, Hungary

*** Correspondence:**Zoárd Tibor Krasznai
[krasznai.zoard@med.unideb.hu](mailto:krasznai.zoard@med.unideb.hu).

# Supplementary Material

**Detailed technical description of the histopathological analysis of the placenta**

After macroscopic examination of the placenta, tissue blocks were selected for further pathological processing. After 24 hours of formalin fixation and paraffin embedding, specimens were sectioned at a thickness of 3 μm and mounted on coated on superfrost slides. Besides standard hematoxylin and eosin stained slides, immunohistological evaluation was performed using the Ventana BenchMark ULTRA IHC/ISH slide staining system (Ventana Medical Systems, Tucson, AZ). The system utilises pre-diluted Liquid Coverslips (catalog #: 5424534001) to prevent drying out of tissues throughout the staining procedure.

**Anti-CD68 staining**

Anti-CD68 staining protocol included online deparaffinization and heat induced epitope retrieval (HIER) on the Ventana BenchMark ULTRA IHC/ISH slide staining system (Ventana Medical Systems, Tucson, AZ). ULTRA Cell Conditioning Solution (ULTRA CC1, catalog #: 5424569001, contains: Tris-based buffer and a preservative) was used as buffer. The time required for epitope retrieval was 30 min at 100°C. Anti-CD68 primary antibody (catalog #: M081401, Dako, Agilent Technologies Company, Santa Clara, CA) was diluted with an antibody diluent (catalog #: 251-018, Thermo Fisher Scientific, Waltham, MA) in 1:1000 ratio. Incubation time was 45 min at 35 °C. Antigen-antibody reactions were visualized with the UltraView DAB IHC Detection kit (catalog #:5269806001, Ventana Medical Systems, Tucson, AZ). The kit detects the primary antibody by a cocktail of enzyme-labeled secondary antibodies (the horse radish peroxidase enzyme is directly conjugated to the secondary antibody). This complex is then visualized with hydrogen peroxidase substrate and DAB chromogen, producing a brown precipitate. The biotin-free chemistry minimizes background staining by eliminating staining of endogenous biotin.

After the staining procedure, slides were removed from the system, dehydrated, and coverslipped for microscopic evaluation.

**Anti-SARS-CoV-2 spike protein staining**

Similarly to the anti-CD68 staining protocol, deparaffinization and HIER was carried out on Ventana BenchMark ULTRA IHC/ISH slide staining system (Ventana Medical Systems, Tucson, AZ), and the ULTRA Cell Conditioning Solution (ULTRA CC1, catalog #: 5424569001, contains: Tris-based buffer and a preservative) was used as buffer. The time required for epitope retrieval was 48 min at 100°C.

Anti-SARS-CoV-2 spike protein (S1) staining was performed using a rabbit antibody from Cell Signalling Technology (E5S3V, catalog#: 99423, Danvers, MA), diluted with an antibody diluent (catalog #: 251-018, Thermo Fisher Scientific, Waltham, MA) in 1:500 ratio. Incubation time was 45 min at 35 °C. Antigen-antibody reactions were visualized with the OptiView DAB IHC Detection Kit (Ventana Medical Systems, Tucson, AZ) as an indirect, biotin-free system for the detection of rabbit primary antibodies. The anti-SARS-CoV-2 spike antibody used in this staining procedure has been confirmed to detect the following SARS-CoV-2 variants: USA-WA1/2020, Alpha (B.1.1.7), Beta (B.1.351), Gamma (P.1), Delta (B.1.617.2), and Omicron (B.1.1.529). The antibody detects full-length (uncleaved) SARS-CoV-2 spike protein, and the fragment corresponding to the S1 domain generated by endogenous protease cleavage. The antibody does not cross-react with spike proteins from other SARS or MERS coronaviruses. After the staining procedure, slides were removed from the system, dehydrated, and coverslipped for microscopic evaluation.
